# Digital Health and Climate Change: Critical Informatics for Change

**DOI:** 10.1055/s-0046-1816587

**Published:** 2026-11-03

**Authors:** Kate Fultz Hollis, Fleur Mougin, Lina F. Soualmia

**Affiliations:** 1 Oregon Health & Science University, Division of Informatics, Clinical Epidemiology, and Translational Data SciencePortlandOregonUSA; 2 Univ. Bordeaux, InsermU1219, BPH, AHeaD teamBordeauxFrance; 3 Univ. Rouen Normandie, Normandie UnivTIBS-LITIS UR 4108F-76000RouenFrance

**Keywords:** Medical Informatics, Health Information Technology, Digital Health, Climate Change, IMIA Yearbook of Medical Informatics

## Abstract

**Objectives:**

To introduce the 2025 International Medical Informatics Association (IMIA) Yearbook of Medical Informatics. The editors of the Yearbook present the contents of 13 sections, and IMIA working group papers and the keynote paper.

**Methods:**

The editorial presents the 2025 IMIA Yearbook whose special theme is “Digital Health and Climate Change”. The section editors introduce the special topic, the survey papers and section best paper overviews. Changes in the Yearbook editorial board are also described.

**Results:**

The IMIA Yearbook 2025 provides different perspectives on current issues on “Digital Health and Climate Change”. Many sections present original work on this year’s theme, and all sections describe notable contributions from 2024 in the various medical informatics specialties covered by the Yearbook.

**Conclusions:**

“Digital Health and Climate Change” is a major issue: while digital technologies have environmental costs, they also can drive environmentally sustainable innovation, and while digital tools can reduce the environmental footprint of the healthcare sector, particularly by reducing CO2 emissions through telemedicine, their technological development also has a negative impact on the planetary health.

## 1. Digital Health and Climate Change

As we were finishing production of the IMIA Yearbook of Medical Informatics in the summer 2025, an extremely intense heatwave swept across the globe. According to research at the University of Maryland, US, the years 2023 and 2024 were the warmest globally since 1850, and the observed increase in the annual area of forest disturbances is due to fires in most climate domains and continents [ 1 ]. Substantial evidence is available that the “ *Earth is warming at an unprecedented rate, and a principal cause is human activity* ” 1 . There are more and more studies about the health effects of climate change. For example, the U.S. journal *Health Affairs* is planning a theme issue on the intersections of climate, health, and equity for early 2026.

For the 34th edition of the IMIA Yearbook, we investigated how climate change problems affect health and look at how digital tools can both minimize and exacerbate global warming.

The Yearbook keynote paper presents this dual nature present in digital tools and the climate. Zerina Lokmic-Tomkins from Monash University contends that *“(d)igital innovation’s promise lies in its immense potential to support global and planetary health through equitable access to healthcare, education, and global economic opportunities. On the other hand, the rapid expansion of digital technology comes at a cost: a deepening the digital divide, an acceleration of environmental destruction, and the spread of misinformation that erodes trust in scientific knowledge* .” Dr. Lokmic-Tomkins outlines many environmental effects of digital health tools that cause harm such as heavy use of data centres to manage medical data and the example of telemedicine (thought to cut travel energy for patient and clinician) infrastructure supporting video consultations, streaming services, cloud storage, and cybersecurity measures requires continuous energy input [ 2 ].

However, as noted many times in the keynote paper, we can encourage and develop better ways to use digital tools to help improve our environment. The conclusion of the paper outlines that while digital technologies have environmental costs, they also can drive environmentally sustainable innovation. Dr. Lokmic-Tomkins suggests that by integrating green practices, such as energy-efficient data management and sustainable ICT device design, we can reduce technology’s environmental footprint while advancing global and planetary health outcomes.

The 2025 sections of the Yearbook include more examples of how climate change can affect digital health. The survey paper of the special topic by Manijeh Berenji *et al.* [ 3 ] examines the role of digital technologies in addressing climate change, exploring different perspectives: mitigation, adaptation, insight, and equity. The authors highlight that current research demonstrates the potential of digital health to enhance the sustainability and inclusivity of healthcare systems, provided that climate considerations are properly integrated.

In the Decision Support survey paper, Titus Schleyer *et al.* [ 4 ] propose advancing healthcare sustainability through clinical decision support. The survey includes many activities with climate change aspects that need to be reviewed including energy, transportation, pharmaceuticals, anesthetic gases, medical devices, and food. All these activities are present in healthcare. The paper ends with important recommendations for sustainability that need to be addressed in healthcare operations.

Section editors for Consumer Health Informatics (CHI) section Pascal Staccini and Annie Lau identify how recent interdisciplinary research integrates digital health, citizen engagement, and behavioral science to address climate-related health challenges and inform mitigation strategies [ 5 ]. The CHI survey paper by Hania Rahimi-Ardabili *et al.* [ 6 ] presents how digital tools may assist in generating and disseminating tailored actionable messages to communities and debunking misinformation, particularly to vulnerable populations . Additionally, technologies that enable visualization, simulation, sensing, and social connection can enhance risk understanding and create opportunities for the adoption of protective behavior among consumers.

Vaibhav Tyagi *et al.* [ 7 ] explore the relationship between health technology and planetary health, and examine whether human and organizational factors (HOFs) were considered in this relationship. This survey for the Human Factors and Organizational Issues (HFOI) section highlights an important point: addressing research gaps and integrating HOFs into future studies will be essential for designing and implementing sustainable healthcare solutions that align with planetary health goals. HFOI section editors Vignesh Subbian and Anthony Solomonides review the 2024 articles that represent diverse methodological approaches, from rigorous application of established human factors frameworks to innovative theoretical developments that expand our understanding of human-technology interactions in healthcare [ 8 ].

In the clinical research informatics (CRI) survey paper, Parveen Kumar and Isabelle de Zegher expand more on technology risks [ 9 ]. Key findings show that, while digital health enhances healthcare adaptability related to climate risks, the size of the impact on environmental resources is uncertain though sufficient to mandate actions. Voluntary mitigation measures are emerging in health-related software development, data lifecycle management, and hardware deployment.

In the Public Health and Epidemiology Informatics (PHEI) survey paper, Torres Santos-Olmo *et al.* [ 10 ] focus on the ambivalent nature of ICTs in healthcare, having both positive and negative impacts. The authors emphasize that ICTs can mitigate the environmental footprint of digital health provided that they comply with responsible and green practices that must be supported by policies.

Thomas Schabetsberger *et al.* [ 11 ] wrote the survey paper for the Clinical Information Systems (CIS) section, which aims to investigate whether and how the areas of health information exchange, care collaboration, and patient engagement can contribute to more sustainable health . The authors show in particular that virtual consultations significantly reduce CO2 emissions. Linda Peute and Romaric Marcilly, editors of this year’s special topic, point out that the best articles they selected more generally emphasize the beneficial role of telemedicine in reducing greenhouse gas emissions [ 12 ].

## 2. Other Highlights of the 34th Edition of the IMIA Yearbook

Although the theme ‘ *Digital Health and Climate Change* ’ appears in many of the section synopses and survey papers, some sections have not selected articles on the theme. In the CRI section, section editors Dipak Kalra, Xavier Tannier, and Pascal Coorevits remark that although this year’s Yearbook focus should ideally highlight environmental issues, they found that too few papers explicitly addressed this perspective within the context of clinical research informatics. For this year in the CRI section, the key themes include artificial intelligence (AI) bias and data representativeness, semantic interoperability and data quality, as well as scalable phenotyping and real-world evidence generation [ 13 ]. The PHEI section editors Brian Dixon and John Holmes also identify AI as a key player in supporting disease surveillance [ 14 ]. Other articles they have selected address communication issues, particularly in combating misinformation or promoting prevention, as well as the modernization of health information systems.

Biosignal and Imaging Informatics (BII) section editors Christian Baumgartner, Leticia Rittner, and Thomas M. Deserno noted that 2024 reflected a maturing BII landscape, wherein computational models are increasingly designed for integration into real-world healthcare settings [ 15 ]. They mention that delineations between signal acquisition, algorithmic modelling, and clinical decision-making continue to dissolve as BII is solidifying its role as a foundational component in the future of intelligent, data-driven medicine. The BII survey paper also further contextualizes the BII field’s trajectory by integrating biosystem identification with biosignal coupling, reinforcing the need for sophisticated models that can capture complex physiological interactions [ 16 ]. This synthesis of methodological rigour and clinical applicability exemplifies the future direction of BII. BII Survey authors Kostoglou and Müller-Putz discuss applications in real-time monitoring, personalized medicine, and clinical diagnostics and illustrate the potential of these techniques in both biomedical research and healthcare.

Sue Feldman and Meryl Bloomrosen, editors of the health information and exchange (HIE) section, note in their 2024 synopsis that studies published in 2024 with research on HIE has reached a higher level of maturity compared to previous years, with an increasing number of international studies [ 17 ]. They also highlight a rising focus on open data standards, the protection of citizens’ rights, and robust data governance—particularly concerning data provenance and control. In their HIE survey paper, Joshua Vest *et al.* [ 18 ] observe that research on HIE adopts diverse perspectives. A significant number of studies treat HIE mainly as a data repository, especially when applying machine learning techniques to HIE-derived data and incorporating social determinants of health. Meanwhile, cybersecurity research within HIE tends to concentrate on technical processes and organizational networks.

Acknowledging that the COVID-19 crisis underscored the importance of detecting emerging terms and their relationship to existing clinical terminology, David Martinez *et al.* [ 19 ] conduct a review of current methods and datasets for identifying infectious diseases in electronic health records, as reported in their natural language processing (NLP) survey. More recent studies, as expected, have focused on developing large language models (LLMs) for this purpose, with reported results demonstrating strong performance across most traditional tasks. NLP section editors Cyril Grouin and Natalia Grabar observe the same trend in their synopsis [ 20 ]. They also emphasize the rise of multi-framework approaches (covering multiple languages, tasks, or models), the use of newspapers as a new source of information, and the growing volume of publications from China—even though English data still dominates.

LLMs are also gaining ground in the field of knowledge representation and management (KRM), as illustrated in the synopsis of this section by Catalina Martínez Costa, Jean Charlet and Licong Cui [ 21 ]. Indeed, the KRM section editors note that they are being applied to enhance ontology development, knowledge graph completion, knowledge engineering, and reasoning. In the KRM survey paper, Gongbo Zhang *et al.* [ 22 ] also report several works that specifically leverage LLMs for continuous knowledge representation learning in particular . Werner Hackl, Sabrina Neururer, and Bernhard Pfeifer, editors of the CIS section, highlight in particular in their synopsis the increasing importance of NLP and AI in enhancing clinical workflows, supporting prediction, and advancing personalization in healthcare [ 23 ].

Cancer informatics has also undergone some transformation with new real-world deployment of digital tools. Section editors Sanjay Aneja and Ravi Parikh remark that while historical cancer informatics research concentrated primarily on algorithmic development and proof-of-concept studies, contemporary efforts prioritize real-world deployment and clinical validation [ 24 ]. The selected papers of 2024 exemplify this paradigm shift, featuring informatics solutions that demonstrate either rigorous prospective evaluation through randomized controlled trials or practical scalability across diverse healthcare environments. Teja Ganta [ 25 ] adds more examples of real-world deployment in oncology for the CI survey where key informatics developments in the fields of health services research and quality improvement highlight how digital health solutions have been implemented in oncology settings to address operational challenges.

New information retrieval tools are being introduced to the Yearbook as Bioinformatics and Translational Informatics (BTI) editors Scott McGrath and Mary Lauren Benton used the relatively new social media Bluesky to find good references to BTI papers [ 26 ]. In addition to their structured PubMed search, McGrath and Benton utilized BlueSky crowdsourcing to identify potentially relevant papers that might have been missed by their traditional search strategy. Overall, they find that the advances in multi-modal data collection and increasingly large datasets lay the groundwork for more complex computational modeling for BTI. Of course any discussion of new tools includes AI tools to discover relevant information. For the BTI survey paper, Alekseyenko *et al.* [ 27 ] provide both positive and negative aspects of generative AI tools: “ *Generative AI tools demonstrate potential in scientific literature synthesis as evidenced by their ability to identify relevant application of data science and informatics in environment and human health applications. Some challenges, such as some omitted references and the need to iterate through the prompts necessitate the need for expert supervision of these tools.”* .

You will find also in this 34th edition of the IMIA Yearbook valuable contributions from several IMIA Working Groups related to the “Digital Health and Climate Change” topic.

## 3. Changes in the Yearbook Editorial Team

As chief co-editor of the IMIA Yearbook, I (Kate Fultz Hollis) have been honored to be part of IMIA Yearbook for seven (7) years and especially grateful for the special leadership of senior editor Brigitte Seroussi when I started and of the current senior editor Lina Soualmia, chief co-editor Fleur Mougin, and best paper editor Adrien Ugon. It is now time for the Yearbook to find a new and I am positive much improved co-editor for many of the Yearbook sections, and I really want to thank all my wonderful section editors for their work on this publication. It is very sad for me to leave a great job as co-chief editor and as always, I look forward to new yearbooks each year. Thank you very much to IMIA past president Christoph U. Lehmann for nominating me for this position, which has been so valuable for me in my informatics career. Continuing with departures, we want to thank very much Christoph Lehmann who is stepping down from Decision Support team. Scott McGrath has done a wonderful job with BTI and he has found great best papers and helped with survey authors for the BTI section for several years. Special thanks also to Leticia Rittner who has been a very valuable member of the BII team for several years.

## Footnotes

1
https://science.nasa.gov/climate-change/
